# Surgical therapy of celiac axis and superior mesenteric artery syndrome

**DOI:** 10.1007/s00423-023-02803-w

**Published:** 2023-01-24

**Authors:** J. P. Jonas, F. Rössler, S. Ghafoor, A. Kobe, T. Pfammatter, C. Schlag, C. A. Gutschow, H. Petrowsky, P. C. Müller, C. E. Oberkofler

**Affiliations:** 1 Department of Surgery and Transplantation, Swiss HPB & Transplant Center Zurich, University Hospital Zurich, Zurich, Switzerland; 2 Department of Diagnostic and Interventional Radiology, University Hospital Zurich, Zurich, Switzerland; 3 Department of Gastroenterology and Hepatology, University Hospital Zurich, Zurich, Switzerland; 4 Vivévis AG–Visceral, Tumor, Robotic Surgery, Clinic Hirslanden, Zurich, Switzerland

**Keywords:** Dunbar, Wilkie, Median arcuate ligament syndrome, Superior mesenteric artery syndrome, Surgery, Failure to treat

## Abstract

**Introduction:**

Compression syndromes of the celiac artery (CAS) or superior mesenteric artery (SMAS) are rare conditions that are difficult to diagnose; optimal treatment remains complex, and symptoms often persist after surgery. We aim to review the literature on surgical treatment and postoperative outcome in CAS and SMAS syndrome.

**Methods:**

A systematic literature review of medical literature databases on the surgical treatment of CAS and SMAS syndrome was performed from 2000 to 2022. Articles were included according to PROSPERO guidelines. The primary endpoint was the failure-to-treat rate, defined as persistence of symptoms at first follow-up.

**Results:**

Twenty-three studies on CAS (*n* = 548) and 11 on SMAS (*n* = 168) undergoing surgery were included. Failure-to-treat rate was 28% for CAS and 21% for SMAS. Intraoperative blood loss was 95 ml (0–217) and 31 ml (21–50), respectively, and conversion rate was 4% in CAS patients and 0% for SMAS. Major postoperative morbidity was 2% for each group, and mortality was described in 0% of CAS and 0.4% of SMAS patients. Median length of stay was 3 days (1–12) for CAS and 5 days (1–10) for SMAS patients. Consequently, 47% of CAS and 5% of SMAS patients underwent subsequent interventions for persisting symptoms.

**Conclusion:**

Failure of surgical treatment was observed in up to every forth patient with a high rate of subsequent interventions. A thorough preoperative work-up with a careful patient selection is of paramount importance. Nevertheless, the surgical procedure was associated with a beneficial risk profile and can be performed minimally invasive.

**Supplementary Information:**

The online version contains supplementary material available at 10.1007/s00423-023-02803-w.

## Introduction

**Fig. 1:**
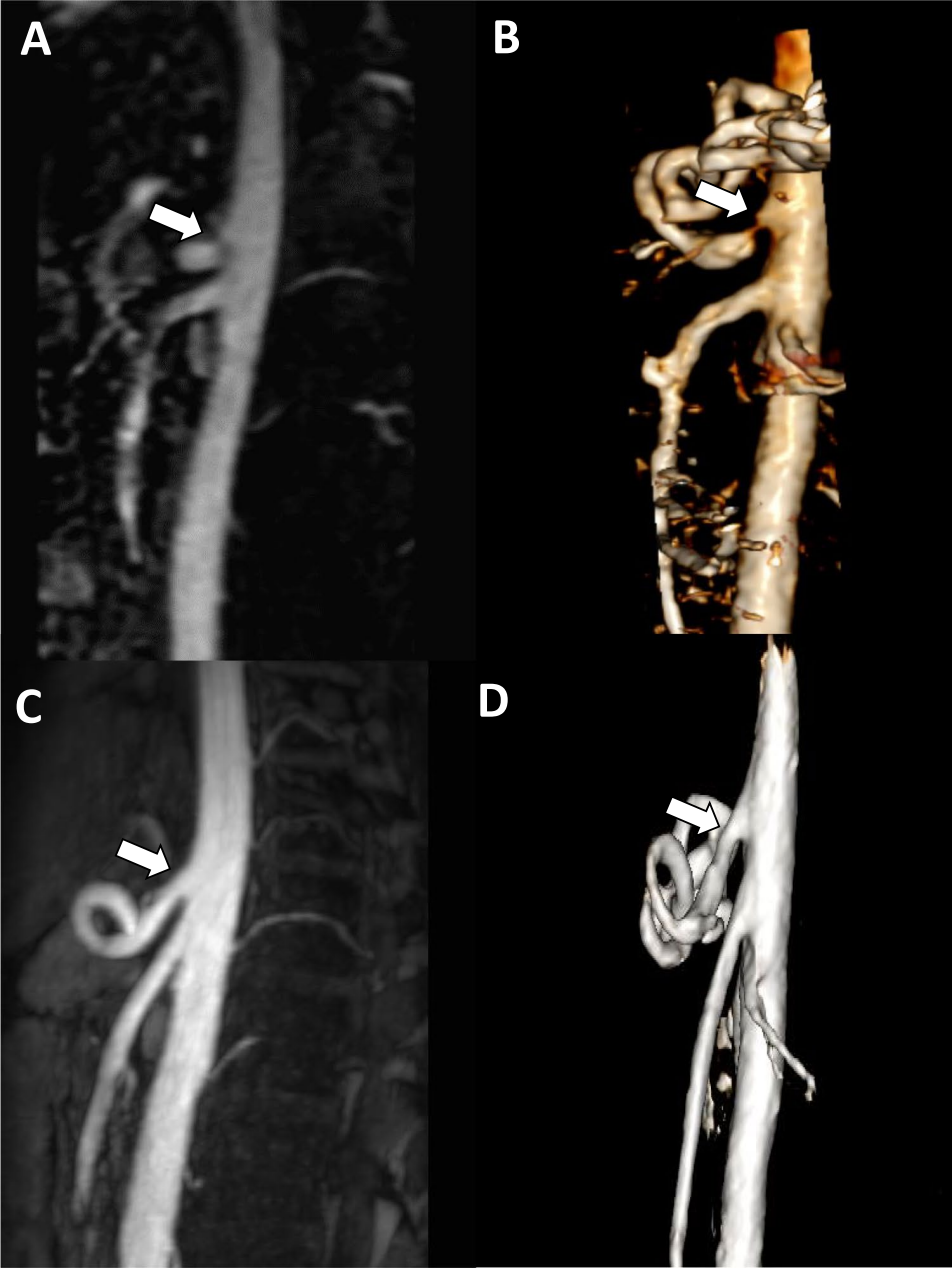
Celiac artery syndrome. Sagittal images (**A**) and 3D reconstruction (**B**) or MR angiography showing severe stenosis of the proximal celiac artery (white arrow in **A** and **B**) due to compression by the median arcuate ligament. Images after surgical release (**C** and **D**) show resolved stenosis (white arrow in **C** and **D**)

Compression syndromes of the celiac artery (CA) or superior mesenteric artery (SMA) are rare conditions with widely varying etiologies and usually require further clinical investigation to properly treat the underlying cause. The best-known representatives of vascular compression of abdominal organs include median arcuate ligament syndrome, also known as Dunbar or celiac artery syndrome (CAS) and superior mesenteric artery syndrome (SMAS), also known as Wilkie syndrome. CAS, was first described by Lipschutz in 1917 using anatomical preparations and later by Harjola in 1963 [1, 2]. Dunbar et al. published a case series of patients treated surgically for CAS in 1965 [3]. The current pathophysiologic understanding of CAS is based on direct external compression of the celiac artery by the median arcuate ligament (Fig. 1). CAS typically occurs more frequently in women (4:1 ratio). A widely accepted hypothesis is that compression of the CA leads to ischemia of the proximal part of the digestive tract. Consequently, food intake leads to increased blood flow demand in the foregut and may exacerbate the symptoms of postprandial pain and involuntary weight loss. However, the exact pathophysiological mechanism remains unclear, and symptoms are often nonspecific. Therefore, patients with CAS are often referred from one specialist to another and undergo extensive diagnostic workup. Therapeutic options primarily include a surgical approach (ligamentum arcuatum release) or an endoluminal approach. Here, usually a stent implantation or balloon angioplasty is performed by interventional radiologists. Sometimes, combined therapy with the common goal of restoring blood flow to the celiac trunk is performed.

**Fig. 2:**
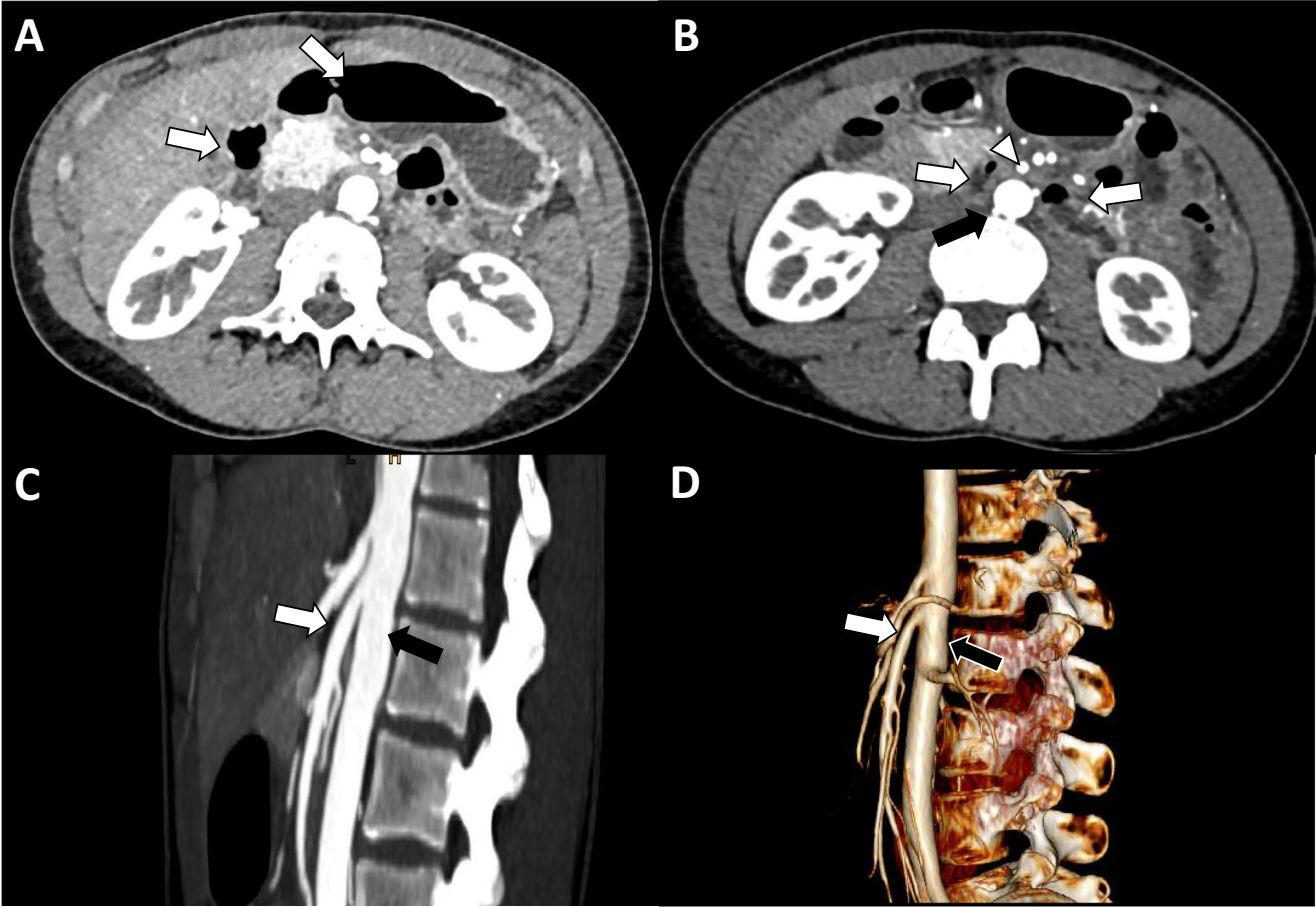
Superior mesenteric artery syndrome. Axial contrast-enhanced CT images at the level of the upper abdomen. **A** Mild distension of the stomach and proximal duodenum (white arrows). **B** Compression of the third portion of the duodenum (white arrows) between the superior mesenteric artery (white arrowhead) and abdominal aorta (black arrow). **C** Sagittal images show reduced angle and distance between the superior mesenteric artery (white arrow) and abdominal aorta (black arrow) (aorto-mesenteric angle of 18°, aorto-mesenteric distance of 5 mm). **D** Sagittal 3D reconstruction again shows the reduced angle between the superior mesenteric artery (white arrow) and abdominal aorta (black arrow)

SMAS was first described by Carl von Rokitansky in 1861 [4], and later a case series of 75 patients was published by Wilkie in 1927 [4]. Here, SMAS refers to the extrinsic compression of the third part of the duodenum by the SMA, causing small bowel obstruction (Fig. 2). The causes of this obstruction may be anatomic such as a low origin of the SMA or an abnormally high ligament of Treitz. As a result of these abnormalities, the aortomesenteric angle encompassing the duodenum along with the adipose tissue (normally between 38 and 65°) may decrease to values as low as 6° [5]. This decreases the available space for the duodenum anterior of the aorta and can potentially cause bowel compression or even compression of the left renal vein [6].

Patients are usually young and female. Symptoms usually entail postprandial pain and vomiting, unintentional weight loss, and rapid onset of postprandial feeling of fullness. A surgical release of the ligament of Treitz or a lateral duodenojejunostomy is recommended if conservative approaches fail. In addition, infrarenal transposition of the SMA is another treatment option. During this procedure, the original set-off of the SMA is ligated and re-anastomosed infrarenally to the anterior aortic wall [5, 7].

To date, the diagnostic workup and optimal treatment strategy for both CAS and SMAS remains unclear. Only a limited group of patients seems to benefit from invasive treatment, and therefore patient selection remains key to successful therapy. However, due to the rarity of these two disorders, available literature consists mainly of case reports and small retrospective case series. The aim of this study was to perform a systematic literature review and summarize the highest level of evidence on CAS and SMAS to present therapeutic recommendations.

## Methods

This systematic review adheres to the PRISMA guidelines [8]. This review was prospectively registered with PROSPERO (International Prospective Register of Systematic Reviews) under study number CRD42021236757 [9].

### Literature search

A comprehensive search of three electronic databases was performed: MEDLINE (via PubMed), Embase, and Cochrane Central Register Database. The following terms were used: “Dunbar,” “Wilkie,” “median arcuate ligament syndrome,” “celiac artery syndrome,” “superior mesenteric artery syndrome,” combined with “therapy,” “treatment,” or “surgery”. The search included publications from 2000 to June 2022, and duplicates were screened by author name, title, and journal. Additionally, the bibliographies of all included studies and existing systematic reviews were searched by hand for appropriate references.

The exact search strategy and search terms can be found in Supplementary Fig. 1.

Two screening phases were performed: [1] title and abstract and [2] full text. Two researchers reviewed the publications and reached consensus to decide on inclusion. Any disagreement was resolved by consulting a third reviewer.

### Eligibility criteria

Inclusion criteria:

1. original articles addressing surgical treatment and outcome of CAS /SMAS;
2. case series of ≥ 5 patients.

The exclusion criteria were.

1. language other than English or German;
2. study not focusing on surgical therapy for CAS or SMAS;
3. published before the year 2000.

### Risk of bias

The quality of included case series articles was assessed by the Robins-I tool*.* This tool assesses risk of bias in non-randomized studies (Supplementary Table 1). Following factors that bear a risk of bias are evaluated and put into a cumulative score: selection of participants, classification of interventions, deviations from main intervention, missing data, or measured outcome variables [10]. Finally, these individual points are put together according to the tool’s manual, and a final evaluation can be made.

### Outcome parameters

The primary endpoint was the failure-to-treat rate, defined as symptom persistence after the first follow-up after surgery. Further, we defined following secondary endpoints: additional interventions for persisting symptoms, perioperative morbidity and mortality graded according to the Clavien–Dindo classification [11], and assessment of quality of life where available. Studies were analyzed whether they provided information on [1] surgical experience for CAS and/or SMAS with number of previous surgeries performed; [2] surgical experience with index procedures (hepato-pancreato-biliary surgery, upper gastrointestinal procedures), and [3] surgeon’s previous experience or training (e.g., fellowships).

### Statistics

All descriptive statistics were performed with SPSS software (SPSS, Inc., Chicago, IL, USA, Version 25). Continuous data are expressed as the median (interquartile range).

## Results

**Fig. 3:**
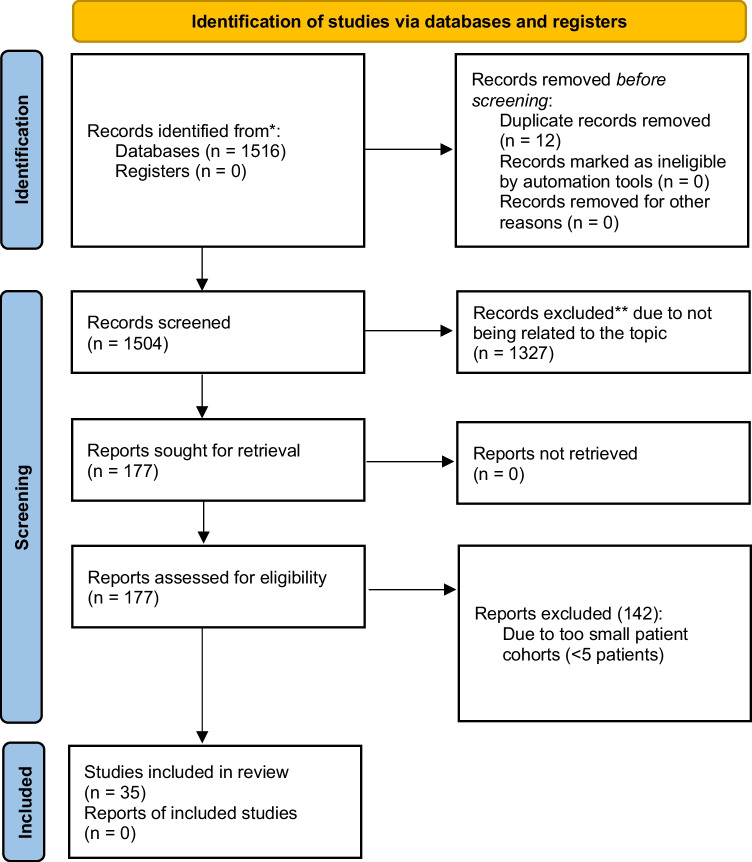
PRISMA workflow diagram 2020

A total of 1516 abstracts were found in the initial search. After removing duplicates and reviewing titles and abstracts, 177 studies and their respective full texts were reviewed for eligibility. As a result, 134 articles were excluded and 35 were included in this systematic review (Table 1A and 1B). The workflow was conducted and documented according to PRISMA guidelines (Fig. 3) [12]. Of the included 35 publications, 24 studies focused on surgical therapy for CAS [13–36] and 11 on SMAS [5, 37–46].

**Table 1:** **A** Overview of included CAS studies. **B** Overview of included SMAS studies

A														
Name	Year	Nr. patients (n)	Gender (f/m)	Age (years)	Follow-up (months)	Diagnostics	Surgical approach (*n*)	Operative time (minutes)	Conversion rate (%)	Intraoperative blood loss (milliliters)	C-D > 3a *	LOS (days)	Failure to treat (%)	
Baccari et al. [20]	2009	16	11/5	52	28	US, CT	Lsc	90	13	50	n/a	3	12,5	
Grotemeyer et al. [21]	2009	18	15/3	46	41	DSA, US	Open	n/a	0	n/a	n/a	12	60	
Roseborough et al. [22]	2009	15	13/2	40	44	DSA, CT	Lsc	189	27	n/a	0	3	40	
Tulloch et al.[23]	2010	8	7/1	45	14	US, CT	Lsc	220	25	50	0	2	50	
Kohn et al. [24]	2011	6	3/3	38	48	US, CT	Lsc (2) and open (4)	n/a	0	n/a	n/a	n/a	0	
Berard et al. [25]	2012	11	9/2	52	35	CT	Lsc	80	18	195	n/a	n/a	9	
Nguyen et al. [26]	2012	5	n/a	32	19	CT	Lsc	120	0	n/a	n/a	4	0	
Do et al. [27]	2013	16	10/6	46	22	US, CT	Lsc (12) a robotic (4)	120	0	n/a	0	1	37	
El-Hayek et al. [28]	2013	15	14/1	34	15	US, CT	Lsc	179	7	217	13	2	25	
Columbo et al. [29]	2015	21	16/5	42	7	US, CT	Lsc	118	0	5	0	2	33	
Klimas et al. [30]	2015	58	47/11	17	62	US, MRI	Lsc	150	3	n/a	n/a	7	7	
Thoolen et al. [31]	2015	9	6/3	46	6	US	Robotic	50	0	50	0	2	55	
Cienfuegos et al. [32]	2017	13	12/1	32	117	CT	Lsc	120	0	20	n/a	3	30	
Duran et al. [33]	2017	31	26/5	45	52	DSA, CT	Open	n/a	0	n/a	13	11	33	
Ho et al. [14]	2017	43	33/10	36	25	DSA, CT	Lsc (38) and open (5)	n/a	2	n/a	0	n/a	62	
Brody et al. [34]	2018	42	41/1	35	28	CT, arteriogram	Lsc	n/a	0	n/a	0	n/a	26	
De'Ath et al. [35]	2018	6	5/1	30	109	CT, MRI	Lsc	137,3	0	110	0	1	0	
Khrucharoen et al. [36]	2018	13	10/3	38	19	US, CT	Robotic	103,5	0	n/a	8	3	23	
Coelho et al. [37]	2020	6	4/2	45	3	CT, MRI	Lsc	93	0	n/a	0	n/a	0	
Fernstrum et al. [38]	2020	27	18/9	49	19	US, CT	Robotic	95	7	100	0	n/a	33	
Khrucharoen et al. [39]	2020	41	28/13	38	16	CT, MRI	Lsc (16) and open (9) and robotic (16)	n/a	5	n/a	n/a	n/a	31	
Sahm et al. [40]	2020	18	12/6	39	5	US, MRI	Lsc	82,7	2	n/a	0	n/a	50	
Pather et al. [41]	2021	100	75/25	38	96	US, CT	Lsc (19) and open (81)	n/a	1	n/a	n/a	4	33	
Kafadar et al. [42]	2022	10	6/4	42	6	CT	Lsc	155	0	150	0	3	n/a	
*n* = 24		*n* = 548	*f *= 421 *m* = 122	Median 39 years	Median 25 months			123 min	Mean 5%	Mean 95 ml	Mean 2%	Median 3 d	Mean 28%	
B														
Name	Year	Nr. patients (n)	Gender (f/m)	Median age (years)	Aortomes. Angle (°)	Follow-up (months)	Diagnostic approach	Surgical approach	Operative time (minutes)	Conversion (%)	Intraoperative blood loss (milliliters)	C-D > 3a *	LOS (days)	Failure to treat (%)
Merrett et al. [5]	2009	8	7/1	27	12	9	CT angio	Lsc	n/a	0	n/a	0	10	0
Lee et al. [17]	2012	15	8/7	28	10	5	Barium, CT angio	Lsc (8) and open (6)	n/a	0	n/a	1	n/a	7
Fang et al. [43]	2014	19	13/6	27	13	6	Upper GI fluoroscopy	Lsc (19) and open (81)	75	0	23	0	8	0
Pottorf et al. [44]	2014	12	7/5	37	n/a	n/a	CT angio	Lsc	n/a	0	n/a	n/a	4	8
Sun et al. [45]	2014	14	11/3	39	n/a	20	US, CT angio	Lsc	119	0	n/a	7	5	21
Chang et al. [46]	2017	18	14/4	31	n/a	26	CT angio	Lsc	144	0	21	6	5	66
Valiathan et al. [47]	2017	6	4/2	20	10	12	CT angio	lsc (3) & open (3)	117	0	n/a	n/a	4	33
Barkhatov et al. [48]	2018	5	5/0	19	10—20	n/a	US, CT angio	lsc	95	0	50	n/a	1	20
Ganss et al. [49]	2018	39	28/11	38	11	47	Barium, CT angio	open	120	0	n/a	5	9	31
Akici et al. [50]	2020	19	13/6	22	10	40	CT angio	lsc	n/a	0	n/a	0	4	n/a
Cienfuegos et al. [51]	2020	13	10/3	24	14.8	94	CT angio	lsc	98	0	n/a	0	4	30
n = 11		*n* = 168	*f* = 120 *m* = 48	median 27 years	median 10°	median 20 months			109 min	mean 0%	mean 31 ml	mean 2%	median 5 d	mean 21%

*LOS*, length of stay; *CT*, computed tomography; *US*, ultrasound; *MRI*, magnetic resonance imaging; *DSA*, digital subtraction angiography; *lsc*, laparoscopic; *min*, minutes; *m*, male; *f*, female; *d*, days; *n*, number

^*^ In analogy to Dindo et al., Ann Surg 2004

LOS = length of stay; CT angio = computed tomography angiography; US = ultrasound; lsc = laparoscopic; min = minutes; m = male; f = female; d = days; n = number;

^*^ in analogy to Dindo et al., Ann Surg 2004

A total of 716 patients were included, of whom 548 were diagnosed and treated surgically for CAS and 168 patients for SMAS. Median age was 39 years [17–52] in CAS and 27 years [19–39] in SMA patients. Of the 548 CAS patients, 421 were female (77%) and in SMAS 120 (71%) were female. The median follow-up was 25 months (3–117) for CAS and 20 months (5–94) for SMAS patients.

Overall, the operating surgeon’s experience was mentioned in 7/35 studies (20%). Six studies (17%) gave information whether surgeons had experience with surgery for CAS and SMAS, although no study gave the exact numbers. One study (3%) described that the performing surgeon had experience in index procedures within the fields of hepato-pancreatico-biliary and upper gastrointestinal surgery. No exact numbers were provided. Four studies (11%) described the training of the surgeon. Here, fellowships with focus on minimal invasive HPB and upper GI are mentioned.

### Diagnosis

Diagnosis of CAS was based on CT scans in 78% of patients. Except for 29 patients (5%), all other CAS patients underwent additional diagnostic imaging such as MRI or ultrasound. In SMAS, 149 (89%) of patients received a CT scan, and in 43%, an additional imaging such as ultrasound, barium swallow scan, or fluoroscopy was performed.

### Treatment

In the CAS group, 27% (*n* = 148) were treated with an open surgical and 73% (*n* = 400) had a minimally invasive approach to release the median arcuate ligament. Regarding surgical access, the laparoscopic approach was used more often 60% (*n* = 321), while the robotic approach was less common with 13% (*n* = 69).In SMAS, 119 (71%) underwent laparoscopic and 48(29%) open surgery. Here, the main procedure performed was either open or minimally invasive: laterolateral duodenojejunostomy or medialization of the duodenum with the aim to pull the duodenum out of the aortomesenteric angle. Interestingly, in our review, only 11 out of 168 patients (7%) underwent a laparoscopic duodenal medialization.

### Perioperative outcomes

Conversion to open surgery was required in 4% (*n* = 23) of all CAS patients. Seventeen patients of 23 (74%) required conversion due to intraoperative bleeding and the rest due to technical difficulties (26%, *n* = 6). Most common bleeding sites were the celiac trunk, left gastric artery, and aorta. Technical reasons for conversion were inadequate exposure and wrong trocar placement. No conversions were reported for the robotic approach. No conversions were reported for SMAS patients.

Intraoperative blood loss was 95 ml (0–217) in CAS patients and 31 ml [21–50] in SMAS patients. Information on postoperative complications was available in only 68% of the included cases (*n* = 634). Postoperative complications occurred in3% of CAS patients (*n* = 15) with a maximal complication Clavien–Dindo grade IIIb. In total, 13 patients (7.7%) who underwent surgery for SMAS had complications ranging from Clavien–Dindo I to V. One death was recorded due to peritonitis following anastomotic leakage of open gastroenterostomy. Median length of stay was 3 days (1–12 days) for CAS and 5 days (1–10 days) for SMAS patients.

### Failure to treat

Overall, the median failure-to-treat rate was 28% in CAS and 21% in SMAS. Within these, consecutive interventions were performed in 47% of CAS and 5% of SMAS patients. These patients underwent further interventions such as angioplasty, re-operation, or endovascular stent placement due to persisting and impairing symptoms.

### Methodological quality

Analysis of the included articles according to the Robins-I tool revealed an overall moderate risk of bias. In total, 2 studies (6%) have a low, 27 a moderate (79%) and 5 (15%) a serious cumulative risk of bias (Supplementary Table 1). A problem frequently seen in these studies is a rudimentary and heterogenous documentation or no structured documentation of the surgical complications or of the required subsequent procedures in cases of symptom persistence.

## Discussion

This systematic review showed a high failure-to-treat rate after surgical therapy for both CAS and SMAS of up to 20– 30%. As a consequence, every second patient for CAS to every 20^th^ patient for SMAS with failure to treat had to undergo consecutive interventions. However, the surgical therapy of both syndromes was associated with a low rate of perioperative complications and a short hospital stay. Current therapeutic standard is a minimal invasive approach either by laparoscopy or robotic-assisted.

**Fig. 4:**
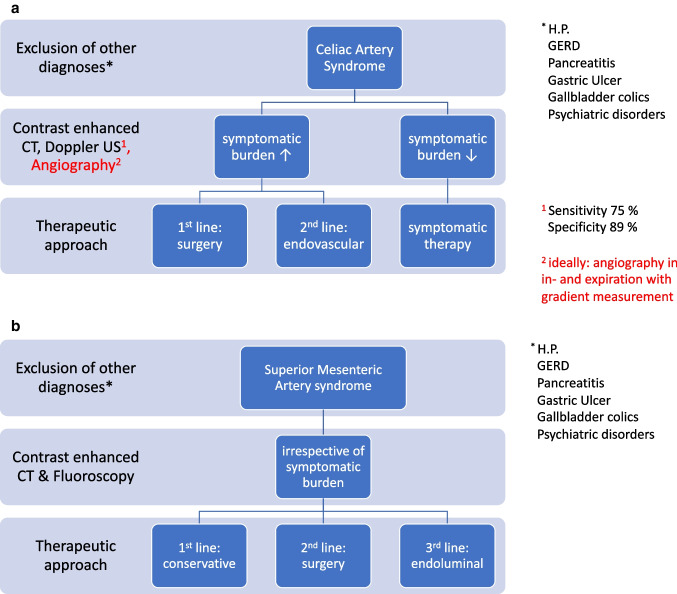
**A** Celiac artery syndrome work-up and treatment algorithm. **B** Superior mesenteric artery syndrome work-up and treatment algorithm. HP, Helicobacter pylori; GERD, gastroesophageal reflux disease; US, ultrasound; contrast enhanced computed tomography (CT)

In most CAS patients, more than one diagnostic modality (e.g., CT and ultrasound/MRI) was performed during the work-up, which is indicative of careful patient selection and a preliminary unclear diagnosis. CAS can be diagnosed by dynamic Doppler ultrasound, which examines blood flow velocity in the celiac axis during inspiration and expiration. Peak systolic velocity during expiratory apnea ultrasound should not exceed 200 m/s in healthy patients and can be considered typical of CAS if symptoms are present [47]. Further, contrast-enhanced CT (ideally performed in end expiration) can be used to highlight the stenotic part of the CA in image reconstruction and help diagnose CAS. Ultimately, patient selection in CAS should focus on clear radiomorphologic evidence of a CA stenosis and especially exclusion of other possible causes to avoid high rates of treatment failure. Therefore, we propose a systematic approach toward the diagnosis of CAS with ideally angiography in in- and expiration with pressure gradient measurement and, after diagnosis, a stepwise therapeutic algorithm (Fig. 4A).

Symptoms of CAS can be diffuse with the most typical being postprandial pain. Nonetheless, other causes such as gastritis, *Helicobacter pylori* infection, and stomach ulcers can cause similar symptoms and need to be ruled out before diagnosis and consecutive surgical treatment of CAS. Low-level evidence suggest that patients suitable for surgical therapy should have no concomitant psychiatric disorders, have a weight loss of less than 9 kg, and be older than 60 years [48]. A recent publication examined a case series of CAS patients for predictive outcome markers after surgery. They found that postexertional pain is a good predictor of surgical outcome (*p* = 0.022) while postprandial vomiting (*p* = 0.046) and pain (*p* = 0.006) before surgery are predictors of poor outcome [13]. For both etiologies, it is critical to identify patients who appear to have multifactorial causes of their symptoms (e.g., psychiatric disorders, gastritis, etc.) and are therefore not ideal candidates for surgical therapy.

In case of SMAS, the correct diagnosis appears to be more comprehensive and intuitive to make. In slim individuals with low body fat and furthermore contrast-enhanced CT evidence of a narrow aortomesenteric angle, the chances of SMAS are high. Likely symptoms are postprandial pain, nausea, and early satiety. Further, fluoroscopy will reveal whether the barium swallow is impeded to pass into the first portion of the jejunum by duodenal compression. Nonetheless, the correct diagnosis still remains one of exclusions due to the rarity of SMAS with a prevalence of 0.1 to 0.3% in the general population [49]. Therefore, malignant and other more frequent diseases have to be excluded. Females between 10 and 40 years old tend to be affected more often [50]. In a first step, adequate conservative therapy consists of improving nutritional status and thereby increasing the aortomesenteric angle via an increase in fatty tissue. A multicentric study showed a success rate of the conservative SMAS treatment in 71% and a relapse rate of 16% [37]. In accordance to this high success rate, patients should undergo surgery only after failed conservative management. In our review, some patients underwent endoluminal therapy (stenting, balloon angioplasty) due to persisting symptoms after failure of conservative and surgical therapy. However, the success rates are difficult to assess from the included studies. A stepwise treatment algorithm considering these therapeutic approaches is proposed in SMAS (Fig. 4B).

According to our review, the preferred surgical approach in ¾ of patients for both syndromes is minimal invasive, with surgeon’s preference either toward robotic or laparoscopic surgery. Conversion rate was very low and mainly due to intraoperative bleeding. A detailed review by Jimenez et al. compared the open with the laparoscopic approach in 400 CAS patients and concluded that symptom relief is better after laparoscopic (96%) than open surgery (78%) [51]. Interestingly, our systematic review shows an overall surgical complication rate within the first 30 postoperative days of only 2.7% in CAS patients with no perioperative mortality. However, in most studies, there was no structured and predefined assessment of complications, leading to a recall bias in these retrospective studies. Of note, the acuity of the documentation of minor complications (C–D grades I–II) is debatable.

Treatment failure after surgery for CAS and SMAS could be explained by different pathological mechanisms of the disease and thus resulting different therapeutic approaches. Correct diagnosis for CAS patients is often long and tedious. However, it is unclear whether reduction in compression of the celiac artery or celiac plexus is more relevant for symptom relief. Furthermore, the extent of CA ligament release is not standardized, and it is conceivable that some surgeons prefer a “light” ligament release with remnant tissue around the CA, whereas other approaches are more aggressive to completely clean the CA with its surrounding nerve and fibrous bundles. Possible causes for treatment failure seem to be vague indications for surgery, neuropathic components of the disease, or a limited preoperative work-up [52]. This indicates that more research on this topic is needed to fully understand the pathophysiology of the disease. A case review of 67 CAS patients that focused on outcome after surgical therapy showed that only 37% of operated patients described a complete resolution of symptoms after surgery; however, still, 56% described a reduction. In this study, two patients required stenting and angioplasty of the CA due to failed decompression with good results [13]. Quality of life was scarcely documented in only 5 of 35 included studies based on objective questionnaires.

Some limitations of the review need to be highlighted. Included studies were all retrospective, and therapeutic recommendations are therefore based on low-level evidence. Further, the majority of articles that were included in this review did not provide any objective assessment of the patients’ symptoms. Of note, only the minority of studies included information about the previous experience and training of the surgeon. Certainly, there is a learning curve for the surgical treatment of CAS and SMAS; however, data on this topic is not available, probably due to the rarity of the diseases. To objectify the success and complication rates of surgery for CAS and SMAS, we suggest that information on the training and expertise of the surgeons should be included. For these reasons, the included studies are of moderate quality with risk of bias.

## Conclusion

In summary, this review highlights a substantial rate of treatment failure in CAS and SMAS patients. Patients should always undergo a structured preoperative diagnostic work-up including contrast-enhanced CT and, ideally, interventional angiography in in- and expiration with pressure gradient measurement and ultrasound for CAS and contrast-enhanced CT and fluoroscopy for SMAS. Before considering surgery, more common differential diagnoses for upper gastrointestinal symptoms need to be excluded. Further, it needs to be highlighted that patients need to be educated preoperatively about possible failure of surgical treatment. As a rule of thumb, if other co-morbidities can be excluded and patients present with a high level of suffering, the success rate of the surgical intervention is deemed better. Minimally invasive surgical treatment remains a valuable first option with a low risk profile in CAS patients. In line, if the SMAS has been detected as causal, a conservative approach including gain of weight has been shown to be a valuable first approach and minimally invasive surgery remains a valuable secondary option with a similar low-risk profile.

### Supplementary Information

Below is the link to the electronic supplementary material.

Supplementary file1 (PDF 590 KB)

Supplementary file2 (PDF 64 KB)

## Data Availability

The data is available in the included studies which were included for this review. Therefore this conclusion has been made by the authors.

## Abbreviations

C-D: Clavien–Dindo
CA: Celiac artery
CAS: Celiac artery syndrome
CT: Computed tomography
EORTC-QLQ-C40: European Organization for Research and Treatment of Cancer Quality of Life of Cancer Patients
GIQLI: The gastrointestinal quality of life index
LOS: Length of stay
MALS: Median arcuate ligament syndrome
MRI: Magnetic resonance imaging
PRISMA: The Preferred Reporting Items for Systematic reviews and Meta-Analyses
SF-12: Short Form-12 questionnaire
SF-36: Short Form-36 questionnaire
SMA: Superior mesenteric artery
SMAS: Superior mesenteric artery syndrome
SPSS: Statistical Package for the Social Sciences
US: Ultrasound
