# Supplementary material for: Surgical therapy of celiac axis and superior mesenteric artery syndrome
Source: Langenbecks Arch Surg. 2023 Jan 24;408(1):59. doi: 10.1007/s00423-023-02803-w (PMC9870837; doi:10.1007/s00423-023-02803-w)
Supplement: Supplementary file 1 — Supplementary file1 (PDF 590 KB) [file 423_2023_2803_MOESM1_ESM.pdf]

Supplementary Figure 1

Cochrane database

Search Name:
Date Run: 25/06/2022 16:27:59
Comment:

ID Search Hits
#1 (dunbar* OR wilkie* OR "median arcuate ligament syndrome" OR "superior mesenteric artery syndrome"):ti,ab,kw (Word variations have been searched) 8
#2 (therap* OR surg* OR radiolog* OR treat* OR decompression* OR laparoscop* OR robotic OR intervention* OR stent* OR dilatation*):ti,ab,kw (Word variations have been searched) 1296759
#3 #1 AND #2 with Publication Year from 2000 to 2022, in Trials 3

Embase database

Embase®

Embase Session Results

No.	Query	Results
#4	#1 AND #2 AND (2000:py OR 2001:py OR 2002:py OR 2003:py OR 2004:py OR 2005:py OR 2006:py OR 2007:py OR 2008:py OR 2009:py OR 2010:py OR 2011:py OR 2012:py OR 2013:py OR 2014:py OR 2015:py OR 2016:py OR 2017:py OR 2018:py OR 2019:py OR 2020:py OR 2021:py) NOT ([animals]/lim NOT [humans]/lim) NOT [conference abstract]/lim	1387
#3	#1 AND #2	2222
#2	'surgery'/exp OR 'therapy'/exp OR 'treatment'/exp OR therap*:ti,ab,kw OR surg*:ti,ab,kw OR radiolog*:ti,ab,kw OR treat*:ti,ab,kw OR decompression*:ti,ab,kw OR laparoscop*:ti,ab,kw OR robotic:ti,ab,kw OR intervention*:ti,ab,kw OR stent*:ti,ab,kw OR dilatation*:ti,ab,kw	17131081
#1	'dunbar syndrome'/exp OR 'celiac artery stenosis'/exp OR dunbar*:ti,ab,kw OR wilkie*:ti,ab,kw OR 'median arcuate ligament syndrome*':ti,ab,kw OR 'superior mesenteric artery syndrome*':ti,ab,kw	2869

Medline search

Ovid® Wolters Kluwer
My Account | Ask a Librarian | Support & Training | University of Zürich | Help | Feedback | Logoff
Search | Journals | Books | Multimedia | My Workspace | Visible Body | What's New

▼ Search History (5) View Saved

# ▲	Searches	Results	Type	Actions	Annotations
1	exp Median Arcuate Ligament Syndrome/ or exp Superior Mesenteric Artery Syndrome/ or (dunbar* or wilkie* or "median arcuate ligament syndrome*" or "superior mesenteric artery syndrome*").ti,ab.	1577	Advanced	Display Results | More	Contract
2	exp General Surgery/ or exp Therapeutics/ or (therap* or surg* or radiolog* or treat* or decompression* or laparoscop* or robotic or intervention* or stent* or dilatation*).ti,ab.	11149014	Advanced	Display Results | More	
3	1 and 2	837	Advanced	Display Results | More	
4	3 not (animals not humans).sh.	826	Advanced	Display Results | More	
5	limit 4 to yr="2000 - 2022	664	Advanced	Display Results | More	

Save Remove Combine with: AND OR
