# Supplementary material for: Surgical therapy of celiac axis and superior mesenteric artery syndrome
Source: Langenbecks Arch Surg. 2023 Jan 24;408(1):59. doi: 10.1007/s00423-023-02803-w (PMC9870837; doi:10.1007/s00423-023-02803-w)
Supplement: Supplementary file 2 — Supplementary file2 (PDF 64 KB) [file 423_2023_2803_MOESM2_ESM.pdf]

Study		Pre-intervention		At intervention	Post-intervention				Overall risk of bias
Name	Year	Bias due to confounding	Bias in selection of participants into the study	Bias in classification of interventions	Bias due to deviations from intended interventions	Bias due to missing data	Bias in measurement of outcomes	Bias in selection of the reported results	low / moderate / serious / critical
Akici et al.	2020	moderate	low	low	low	moderate	low	low	moderate
Baccari et al.	2009	moderate	moderate	low	low	low	low	low	moderate
Barkhatov et al.	2018	low	low	low	low	moderate	low	low	moderate
Berard et al.	2012	low	low	low	low	serious	low	low	serious
Brody et al.	2018	low	low	low	low	moderate	low	low	moderate
Chang et al.	2017	low	moderate	low	low	moderate	moderate	low	moderate
Cienfuegos et al.	2017	low	low	low	low	moderate	low	low	moderate
Cienfuegos et al.	2020	low	low	moderate	low	moderate	low	low	moderate
Coelho et al.	2020	low	moderate	moderate	low	serious	low	low	serious
Columbo et al.	2015	low	low	low	low	serious	low	low	serious
De'Ath et al.	2018	low	low	low	low	low	low	low	low
Do et al.	2013	low	low	moderate	low	moderate	low	low	moderate
Duran et al.	2017	low	low	moderate	low	serious	low	low	serious
El-Hayek et al.	2013	low	low	low	low	moderate	moderate	low	moderate
Fang et al.	2014	low	low	moderate	low	moderate	low	low	moderate
Fernstrum et al.	2020	low	low	low	low	serious	low	low	serious
Ganss et al.	2018	low	low	low	low	moderate	low	low	moderate
Grotemeyer et al.	2009	low	low	moderate	low	moderate	low	low	moderate
Ho et al.	2017	low	moderate	moderate	low	moderate	moderate	low	moderate
Khrucharoen et al.	2018	low	low	low	low	low	low	low	low
Khrucharoen et al.	2020	low	low	low	low	moderate	low	low	moderate
Klimas et al.	2015	low	low	low	low	moderate	low	low	moderate
Kohn et al.	2011	low	low	moderate	low	moderate	low	low	moderate
Lee et al.	2012	moderate	moderate	low	low	moderate	moderate	low	moderate
Merrett et al.	2009	low	low	low	low	moderate	low	low	moderate
Nguyen et al.	2012	low	low	low	low	moderate	low	low	moderate
Pather et al.	2021	low	moderate	moderate	low	moderate	moderate	low	moderate
Pottorf et al.	2014	low	low	low	low	moderate	low	low	moderate
Rosenborough et al.	2009	low	low	low	low	moderate	low	low	moderate
Sahm et al.	2020	low	moderate	low	low	moderate	low	low	moderate
Sun et al.	2014	low	low	moderate	low	moderate	low	low	moderate
Tholeen et al.	2015	low	low	low	low	moderate	low	low	moderate
Tulloch et al.	2010	low	moderate	low	low	moderate	low	low	moderate
Valiathan et al.	2017	low	moderate	low	low	moderate	low	low	moderate
